# Thyroid function and liver fibrosis: FT3 levels are inversely and independently correlated with enhanced liver fibrosis score in solid organ transplant patients with dysglycemia

**DOI:** 10.3389/fimmu.2025.1726617

**Published:** 2026-01-08

**Authors:** Claudia Leanza, Maria Ausilia Giusti, Vitale Miceli, Giovanni Zito, Rosaria Tinnirello, Gioacchin Iannolo, Antonio Galante, Fabrizio Emanuele, Marco Amato, Giovanna Lo Iacono, Vincenzina Lo Re, Salvatore Gruttadauria, Aldo Eugenio Calogero, Massimo Pinzani, Alessandro Mattina

**Affiliations:** 1 UPMC Italy, Palermo, Italy; 2 Department of Clinical and Experimental Medicine, University of Catania, Catania, Italy; 3 Diabetes Service, IRCCS ISMETT, Palermo, Italy; 4 Department of Research, IRCCS ISMETT, Palermo, Italy; 5 Libera Università degli Studi di Enna “Kore”, Department of Medicine and Surgery, Enna, Italy; 6 Abdominal Center Department, IRCCS ISMETT, Palermo, Italy; 7 Neurology Service, IRCCS ISMETT, Palermo, Italy; 8 Department of Surgery and Medical and Surgical Specialties, University of Catania, Catania, Italy; 9 Institute for Liver and Digestive Health, Division of Medicine, University College London, Royal Free Hospital, London, United Kingdom

**Keywords:** solid organ transplantation, FT3, ELF score, MASLD/MASH, fibrosis, dysglycemia, immunosuppression, liver stiffness

## Abstract

**Background:**

Solid organ transplantation (SOT) is frequently complicated by dysglycemia and metabolic dysfunction–associated steatotic liver disease (MASLD), conditions that accelerate the development of liver fibrosis. Given the recognized thyroid–liver crosstalk, we investigated the association between thyroid function and the enhanced liver fibrosis (ELF) score in SOT recipients with diabetes or prediabetes.

**Methods:**

Seventy-one adult SOT recipients with diabetes or prediabetes, with ultrasound evidence of liver steatosis and/or a FIB-4 > 1.3, underwent standardized clinical phenotyping, biochemical profiling, thyroid hormone measurements, ELF testing, and liver stiffness measurement (LSM). Associations with ELF were assessed using correlation analyses and multivariable linear regression adjusted for age, sex, body mass index (BMI), transaminases, glycated haemoglobin, creatinine, haemoglobin, albumin, immunosuppressive drugs, glucagon-like peptide-1 receptor agonist (GLP-1RA) therapy, and transplanted organ type.

**Results:**

Participants had a mean age of 63.1 ± 9.5 years and BMI of 27.8 ± 4.8 kg/m². Mean ELF was 9.21 ± 1.00 (low risk <9.8: 70%; intermediate 9.8–11.3: 27%; high ≥11.3: 3%). ELF correlated positively with age (r=0.43, p=0.0002), aspartate aminotransferase (AST; r=0.50, p<0.0001), alanine aminotransferase (ALT; r=0.33, p=0.0059) and creatinine (r=0.39, p=0.0009), and inversely with haemoglobin (r=−0.39, p=0.0009), albumin (r=−0.38, p=0.0024), controlled attenuation parameter (CAP) (r=−0.29, p=0.0171). Among thyroid variables, free triiodothyronine (FT3) correlated inversely with ELF score (r=−0.45, p=0.0003), while TSH and FT4 showed no significant association with ELF score (r=0.00, p=0.9859; r=-0.5, p=0.6891). In multivariable analysis (R²=0.67; p=0.0002), lower FT3 (β=−0.611 ± 0.288; p=0.0404) and age (β=0.029 ± 0.012; p=0.0304) remained independently associated with higher ELF. No association was found between ELF and LSM.

**Conclusions:**

In SOT recipients with dysglycemia lower FT3 levels were independently associated with increased ELF scores. This finding suggests a potential link between subtle variations in thyroid function and markers of fibrogenic activity in metabolically vulnerable transplant recipients. Prospective studies are warranted to elucidate the causal directionality of this association and its clinical relevance.

## 1 Introduction

Solid organ transplantation (SOT) is a definitive and life-saving treatment for patients with end-stage organ failure (1, 2). Metabolic complications affect more than 50% of SOT recipients and include post-transplant diabetes mellitus (PTDM), metabolic dysfunction–associated steatotic liver disease (MASLD), dyslipidaemia, arterial hypertension and obesity (3). These disorders largely result from the shift to a pro-anabolic state after transplantation, often compounded by lifestyle changes and weight gain, as well as by the distinct metabolic effects of immunosuppressive agents (3–6).

MASLD, defined by the presence of hepatic steatosis in combination with at least one metabolic risk factor, represents a spectrum ranging from simple steatosis to steatohepatitis, fibrosis, and cirrhosis, with potential progression to hepatocellular carcinoma (7, 8). Globally, MASLD affects 25–38% of adults, with prevalence reaching up to 70% among individuals with type 2 diabetes mellitus (T2DM), reflecting shared pathogenic mechanisms such as insulin resistance and obesity that are consistently observed across diverse populations (8–11). Recurrent MASLD occurs in approximately 35–49% of patients undergoing liver transplantation for metabolic cirrhosis, while *de novo* MASLD has been described in 18–78% of recipients transplanted for other indications (12–14). Diabetes, whether pre-existing or emerging after transplantation, represents a major determinant of both recurrent and *de novo* MASLD/MASH, and is strongly associated with accelerated fibrosis progression (15).

The Enhanced Liver Fibrosis (ELF) test is a blood-based biomarker that provides the ELF score, with excellent diagnostic accuracy for detecting advanced fibrosis in MASLD and for guiding risk stratification. It is based on the combined measurement of hyaluronic acid (HA), tissue inhibitor of metalloproteinase-1 (TIMP-1), and the N-terminal propeptide of collagen type III (PIIINP) (16). Some evidence in literature supports the validity of the ELF test also in liver diseases other than ALD and MASLD, such as viral hepatitis and other chronic liver diseases of mixed etiology, as demonstrated by a 2023 meta-analysis (17). Regarding liver transplant patients, a prospective study evaluated the accuracy of the ELF test in a cohort of 87 transplanted patients, comparing it with liver biopsy and ultrasound-based methods (18). The ELF test demonstrated a diagnostic performance comparable to that of ultrasound techniques, with similar AUROC values for the diagnosis of significant fibrosis and cirrhosis, suggesting that the ELF test can also be used in post-transplant follow-up, while taking into account that the pathogenesis of fibrosis in this setting may be influenced by immunological mechanisms and graft rejection.

Thyroid and liver have a complex, bidirectional relationship. Thyroid hormones (TH) regulate hepatic glucose and lipid metabolism, whereas liver dysfunction can influence circulating TH levels and their actions on peripheral tissues (19). Both overt and subclinical hypothyroidism are associated with an increased risk and greater severity of MASLD and metabolic dysfunction–associated steatohepatitis (MASH), mediated by systemic inflammation, oxidative stress, and metabolic dysregulation (20–24). Hypothyroidism, even in its subclinical form, reduces energy expenditure, promotes visceral fat accumulation, atherogenic dyslipidaemia, and insulin resistance, and further disrupts hepatic glucose and lipid metabolism, thereby fostering steatosis, fibrogenesis, and ultimately the development and progression of MASLD (23, 25–27). In MASH, impaired thyroid hormone receptor beta (THR-β) signalling has been observed, leading to reduced hepatic thyroid hormone activity (19, 23, 28, 29). In advanced fibrosis, intrahepatic hypothyroidism has been described, resulting from repair-associated alterations in hepatic deiodinase expression that increase thyroxine (T4) conversion to reverse triiodothyronine (rT3) while reducing triiodothyronine (T3) availability (30). This condition may also occur in individuals with euthyroid status (30, 31). Recognition of this pathophysiological mechanism has contributed to the USA Food and Drug Administration’s approval of resmetirom, a liver-directed thyroid hormone receptor-beta (THR-β) agonist, for the treatment of adult non-cirrhotic MASH with moderate to advanced fibrosis (32).

Solid organ transplant recipients represent a rapidly expanding population due to improved long-term patient and graft survival, characterized by distinctive immunological, pharmacological, and metabolic exposures that are not shared by general or non-transplant diabetic populations. The METHEORIT study is a single-centre, prospective observational cohort study including 71 organ transplant recipients with diabetes or prediabetes, followed for 12 months. The study investigates hepatic steatosis and fibrosis using transient elastography and serum biomarkers, with a focus on the combined impact of diabetes and immunosuppressive therapy on MASLD/MASH risk, as well as the underlying metabolic mechanisms.

The present study reports an exploratory analysis of baseline (T0) data from the METHEORIT cohort, examining the relationship between ELF score and thyroid function. The primary hypothesis was that the FT3–fibrosis association, well established in the general population, is preserved in SOT recipients despite substantial metabolic, pharmacological, and immunological complexity. This observation may potentially underscore the influence of thyroid hormones on the fibrogenic evolution of post-transplant MASLD.

## 2 Materials and methods

### 2.1 Patient population

Between September and December 2024, seventy-one consecutive patients were recruited at the Diabetes Outpatient Clinic of IRCCS ISMETT - UPMC Italy, a tertiary referral transplant centre. The ISMETT - UPMC Diabetes Service provides specialized metabolic care for transplant recipients. Eligible participants were aged ≥18 years, had a diagnosis of diabetes mellitus or prediabetes [impaired glucose tolerance (IGT) and/or impaired fasting glucose (IFG)], and were receiving maintenance immunosuppressive therapy after solid organ transplantation (kidney, pancreas, liver, heart, lung, or combined). Immunosuppressive regimens included calcineurin inhibitors (cyclosporine, tacrolimus), antiproliferative agents (azathioprine, mycophenolate), mammalian/mechanistic target of rapamycin (mTOR) inhibitors (sirolimus, everolimus), corticosteroids, or combination therapy. Additional inclusion criteria were ultrasound evidence of hepatic steatosis and/or a FIB-4 score >1.3.

Exclusion criteria included the presence of non-metabolic associated liver diseases, history of alcohol abuse or current alcohol consumption or other post-transplant conditions associated with liver diseases, decompensated liver cirrhosis, any liver condition that could interfere with elastography assessment, pregnancy or breastfeeding, and inability to provide informed consent due to incapacity or unconsciousness.

The study was conducted in accordance with the Declaration of Helsinki and approved by the Institutional Review Board of IRCCS ISMETT (IRRB-42-23). All participants provided written informed consent before enrolment.

### 2.2 Diagnosis of diabetes, prediabetes, metabolic syndrome and others clinical conditions

Diabetes mellitus was defined as a prior diagnosis by another physician, current antidiabetic treatment, or a new diagnosis established at our centre according to the American Diabetes Association (ADA) guidelines (33): HbA1c ≥ 6.5% (48 mmol/mol) confirmed by repeat testing, fasting plasma glucose ≥ 126 mg/dL on two separate occasions, 2-hour plasma glucose ≥ 200 mg/dL during an oral glucose tolerance test (OGTT), or random plasma glucose ≥ 200 mg/dL in the presence of classic hyperglycaemic symptoms. Prediabetes was defined as impaired fasting glucose (100–125 mg/dL), impaired glucose tolerance (2-hour plasma glucose 140–199 mg/dL during OGTT), or HbA1c 5.7–6.4% in the absence of values diagnostic for overt diabetes.

Regarding PTDM diagnosis, to minimize false positives related to acute post-surgical stress and immunosuppressive treatment, the diagnosis was only confirmed after a stabilization period of at least three months following transplantation.

Metabolic syndrome was defined as the presence of three or more of the following metabolic abnormalities: a waist circumference of more than 102 cm in men and 88 cm in women; serum triglycerides level of 150 mg/dL or greater; reduced high-density lipoprotein cholesterol, less than 40 mg/dL in men or less than 50 mg/dL in women; elevated fasting glucose of 100 mg/dL or greater; systolic blood pressure > 130 mmHg, or diastolic blood pressure > 85 mmHg.

The presence of coronary artery disease, cerebrovascular disease, and renal impairment was evaluated using data from the medical records, based on prior diagnoses established by the relevant specialists.

### 2.3 Assessment of liver stiffness

Liver stiffness was assessed using FibroScan^®^ Expert 630 (SmartExam software FS 4.1.5.1 - Echosens, France), a device based on vibration-controlled transient elastography (VCTE). The system measures liver stiffness (liver stiffness measurement, LSM) at 50 Hz and also provides the controlled attenuation parameter (CAP) at 3.5 MHz to quantify liver steatosis (34). An M+ or XL+ probe was used according to the manufacturer’s instructions, based on the patient’s body composition. All measurements were performed by the same certified operator after at least 4 hours of fasting, to ensure the validity of the measurements.

### 2.4 Blood biochemistry and endocrine assessments

Participants fasted for at least 10 hours overnight before venous blood samples were collected between 7:00 and 10:00 a.m. Serum concentrations of free triiodothyronine (FT3), free thyroxine (FT4), and thyroid-stimulating hormone (TSH) were measured using an automated clinical chemistry analyser (Dimension VISTA 1500, Siemens Healthineers, Erlangen, Germany). The reference ranges for FT3, FT4, and TSH were 1.80–4.20 pg/mL, 0.89–1.76 ng/dL, and 0.400–4.000 µIU/mL, respectively.

Plasma glucose, serum total cholesterol (TC), triglycerides (TG), low-density lipoprotein cholesterol (LDL-C), high-density lipoprotein cholesterol (HDL-C), creatinine, as well as liver and pancreatic function tests (alanine aminotransferase, aspartate aminotransferase, γ-glutamyltransferase, amylase, lipase) were determined using the same analyser (Dimension VISTA 1500, Siemens Healthineers).

Glycated haemoglobin (HbA1c) was measured by high-performance liquid chromatography (Bio-Rad D-10, Hercules, CA, USA). Serum insulin and C-peptide were quantified using the LIAISON XL system (DiaSorin, Saluggia, Italy). Complete blood counts were performed with the Sysmex XN-3000 (Sysmex, Kobe, Japan).

All analyses were performed at a single central laboratory operating under ISO 9001:2015 certification.

### 2.5 Plasma sample preparation and enhanced liver fibrosis test

Blood samples were collected in EDTA tubes and centrifuged at 3500 rpm for 10 minutes to separate plasma. Plasma aliquots were stored at –80°C until analysis, then thawed and gently mixed prior to use. The ELF test is a non-invasive, commercially available blood assay that quantifies HA, PIIINP, and TIMP-1, three biomarkers directly involved in hepatic fibrosis (16). The assay (Siemens Immunoassay Reagent/Calibrator Kit Atellica IM ELF, cat. no. 11208118) was performed according to the manufacturer’s instructions on an Atellica IM 1600 analyser (Siemens Healthineers). Plasma biomarker concentrations were expressed in ng/mL and used to calculate the ELF score according to the following formula: ELF score = 2.278 + 0.851 ln[HA] + 0.751 ln[PIIINP] + 0.394 ln[TIMP-1]. Patients were classified according to their risk of disease progression, based on ELF score thresholds: < 9.80 (low), ≥ 9.80 to < 11.30 (medium), and ≥ 11.30 (high) (8, 35).

### 2.6 Assessment of other liver fibrosis and steatosis scores

FIB-4 index was calculated from routinely collected biochemical blood tests using the following formula: (Age [years] × AST [U/L])/(Platelet count [×10^9^/L] × √ALT [U/L]) (36). Hepatic Steatosis Index (HSI) was calculated as follows: HSI = 8 × [AST (U/L)/ALT (U/L)] + BMI (kg/m²) + 2 (if diabetes) + 2 (if female) (37). The NAFLD Fibrosis Score (NFS) was assessed using the following formula: NFS = –1.675 + 0.037 × Age (years) + 0.094 × BMI (kg/m²) + 1.13 (if diabetes or impaired fasting glucose) + 0.99 × [AST (U/L)/ALT (U/L)] – 0.013 × Platelets (10³/µL) – 0.66 × Albumin (g/dL) (38). The NAFLD Liver Fat Score (NAFLD-LFS) was calculated as: NAFLD-LFS = –2.89 + 1.18 × Metabolic Syndrome (0/1) + 0.45 × Diabetes (0/1) + 0.15 × Insulin (mU/L) + 0.04 × AST (U/L) – 0.94 × ALT/AST ratio (39).

### 2.7 Statistical analysis

The baseline characteristics of the patients were described using measures of central tendency (mean and median) and dispersion (standard deviation and interquartile range) for numerical variables, while categorical variables were summarized using contingency tables of absolute and relative frequencies. Variable distributions were graphically assessed using histograms and boxplots to evaluate distributional shape, normality assumptions, and potential outliers.

The relationships of the ELF score and its components (HA, PIIINP, and TIMP-1) with demographic, metabolic, and transplant-related parameters were assessed using Pearson’s correlation coefficient and scatterplots for numerical variables, whereas for categorical variables Student’s t-test was applied. Associations between categorical covariates were examined using McNemar’s test.

Data analysis was performed using univariate and multivariate linear regression models. Potential predictors included in the multivariate model were free triiodothyronine (FT3), age, body mass index (BMI), use of immunosuppressive drugs (tacrolimus, mycophenolate, everolimus), use of GLP-1 receptor agonists, aspartate aminotransferase (AST), alanine aminotransferase (ALT), glycated haemoglobin (HbA1c), serum creatinine, haemoglobin, albumin, sex, and type of transplanted organ.

To assess the potential impact of modeling adjustments on thyroid–liver associations, sensitivity analyses were performed excluding HbA1c and/or GLP-1RA exposure from the multivariable models (reported in the **Supplementary Material**).

Results were considered statistically significant at two-sided p-values <0.05. All statistical analyses were conducted using RStudio version 2025.05.1 (Build 513) within the R environment (R Core Team, 2025; R Foundation for Statistical Computing, Vienna, Austria).

## 3 Results

### 3.1 Patients characteristics

Patients’ clinical, biochemical, pharmacological and transplant-related characteristics are presented in **Table 1** and **Table 2**. Patients were predominantly male (80.3%), with females accounting for 19.7% of the cohort. The mean age was 63.15 ± 9.5 years. The mean BMI was 27,76 ± 4,8 kg/m^2^, while mean waist and hip circumferences were 100.23 ± 13.7 cm and 101.02 ± 10.4 cm, respectively.

**Table 1:** Demographic and clinical characteristics [n (%), mean ± SD or median (IQR)].

Demographic and clinical characteristics	All subjects
Demographic and anthropometric characteristics	
n	71
Age, years	63.15 ± 9.5
Gender (1=men), n (%)	57 (80.3)
Weight, kg	77.51 ± 14.1
BMI, kg/m^2^	27.76 ± 4.8
Waist circumference, cm	100.23 ± 13.7
Hip circumference, cm	101.02 ± 10.4
Comorbidities and cardiometabolic characteristics	
Prediabetes (IFG or IGT), n (%)	6 (8.5)
Pre-existing diabetes mellitus, n (%)	22 (31)
Post-transplant diabetes mellitus, n (%)	43 (60.6)
Metabolic syndrome, n (%)	30 (42.3)
Dyslipidaemia, n (%)	43 (60.6)
Hypertension, n (%)	57 (80.3)
Coronary heart disease, n (%)	18 (25.4)
Cerebrovascular disease, n (%)	7 (9.9)
Nonsmoker, n (%)	49 (69)
Smoker, n (%)	1 (1.4)
Former smoker, n (%)	16 (22.5)
Renal failure, n (%)	49 (69)
Organ(s) transplanted and outcomes	
Kidney, n (%)	37 (52.1)
Liver, n (%)	21 (29.6)
Lung, n (%)	1 (1.4)
Pancreas, n (%)	1 (1.4)
Heart, n (%)	9 (12.7)
Kidney-pancreas, n (%)	1 (1.4)
Kidney-liver, n (%)	1 (1.4)
Graft rejection history, n (%)	20 (28.2)
Time since transplantation, months	83 (39-183)
Immunosuppressant regimen	
Tacrolimus, n (%)	59 (83.1)
Mycophenolate, n (%)	49 (69)
Steroid, n (%)	33 (46.5)
Cyclosporin, n (%)	2 (2.8)
Everolimus, n (%)	10 (14.1)
Therapy	
Metformin, n (%)	27 (39.1)
Sulfonylureas, n (%)	2 (2.8)
GLP1RA, n (%)	19 (27.5)
SGLT2-i, n (%)	16 (23.2)
DPP4-i, n (%)	1 (1.4)
Fast-acting insulin, n (%)	16 (23.2)
Long-acting insulin, n (%)	29 (42)
β-blockers, n (%)	33 (46.5)
ACEi, n (%)	26 (36.6)
ARBs, n (%)	16 (22.5)
Diuretics, n (%)	17 (23.9)
Calcium channel blockers, n (%)	25 (35.2)
α-blockers, n (%)	20 (28.2)
ASA, n (%)	40 (56.3)
Statins n (%)	42 (59.2)

BMI, body mass index; IFG, impaired fasting glucose; IGT, impaired glucose tolerance; GLP1RA, glucagon-like peptide-1 receptor agonist; SGLT2-i, sodium-glucose cotransporter-2 inhibitor; DPP4-i, dipeptidyl peptidase-4 inhibitor; ACEi, angiotensin-converting enzyme inhibitor; ARBs, angiotensin II receptor blockers; ASA, acetylsalicylic acid.

**Table 2:** Main biochemical parameters [mean ± SD or median (IQR)].

Variables	All subjects
Glycaemia, mg/dl	118 ± 22
Glycated haemoglobin, mmol/mol	43 (38-48)
C-peptide, ng/mL	2.51 (1.88-3.57)
Creatinine, mg/dL	1.23 (0.94-1.5)
Microalbuminuria, mg/L	25.35 (8.62-76)
Alkaline phosphatase, U/L	83.5 (67-127.8)
Total cholesterol, mg/dL	152.7 ± 41
LDL cholesterol, mg/dL	74 (61-100)
HDL cholesterol, mg/dL	57.8 ± 15.6
Triglycerides, mg/dL	115 (96-151)
Amylase, U/L	31 (21-39.75)
Lipase, U/L	37 (27.8-56.3)
Haemoglobin, g/dL	12.9 ± 1.7
WBC, x10³/µL	6.74 ± 2.5

LDL, low-density lipoprotein; HDL, high-density lipoprotein; WBC, white blood cells.

The majority of patients (37; 52.1%) received a kidney-only transplant, 21 (29.6%) underwent liver-only transplantation, 9 (12.7%) received a heart transplant, and the remaining 4 patients underwent lung, pancreas, or combined transplantation. Among liver transplant recipients, the most common indications for transplantation were MASLD-related cirrhosis and viral liver disease (mainly HCV infection). Among kidney recipients, the leading causes were chronic kidney disease of undetermined etiology, polycystic kidney disease, and primary glomerulopathies. Among heart recipients, indications were primarily dilated cardiomyopathy and end-stage heart failure. One lung recipient underwent transplantation for severe panlobular emphysema. Most patients received tacrolimus (83.1%) and mycophenolate (69.0%) as maintenance immunosuppressive therapy. Steroids were used in 46.5% of cases, whereas everolimus and cyclosporine were prescribed less frequently (14.1% and 2.8%, respectively). All patients had stable immunosuppressive therapy levels for at least one month prior to enrolment. Twenty patients (28.2%) experienced acute graft rejection and the median post-transplant duration was 83 months (IQR 39–183), corresponding to approximately seven years after transplantation.

Pre-existing diabetes mellitus was present in 31% of patients, while 60.6% developed post-transplant diabetes mellitus. A prediabetes status (IFG or IGT) was identified in 6 patients. The median value of glycated haemoglobin of our patients was 43 mmol/mol (38–48) [6.08% (5.62-6.54)]. Metabolic syndrome was present in 42.3% of patients, with more than 80% having hypertension, 25.4% a history of coronary heart disease, and approximately 10% a history of cerebrovascular disease. Only one patient was an active smoker, 69% were non-smokers, and 22.5% were former smokers.

Among diabetic patients, 39.1% were receiving metformin, 27.5% glucagon-like peptide-1 receptor agonists (GLP-1RA), and 23.2% sodium-glucose cotransporter-2 inhibitors (SGLT2i). Only two patients (2.8%) and one patient (1.4%) were treated with sulfonylureas and dipeptidyl peptidase-4 inhibitors (DPP4i), respectively. About 23% of patients were on fast-acting insulin, while 42% were on long-acting insulin. Among antihypertensive agents, β-blockers were the most frequently prescribed (46.5%), followed by angiotensin-converting enzyme inhibitors (ACEi, 36.6%) and calcium channel blockers (35.2%). α-blockers (28.2%), diuretics (23.9%), and angiotensin II receptor blockers (ARBs, 22.5%) were used less frequently. In addition, acetylsalicylic acid (ASA) and statins were commonly prescribed in our cohort (56.3% and 59.2%, respectively).

All patients had FT3 and FT4 values within the normal range. Altered TSH levels were observed in 9% of cases (6% >4 µIU/mL and 3% <0.4 µIU/mL). Eight patients (11.3%) had hypothyroidism, treated with levothyroxine, and one patient had hyperthyroidism, treated with methimazole, all maintaining biochemical euthyroidism (**Table 3**).

**Table 3:** Thyroid function [n (%), mean ± SD or median (IQR)].

Thyroid function	All subjects
Thyroid hormones	
TSH, µUI/ml	1.56 (1.08-2.39)
FT3, pg/ml	2.73 ± 0.5
FT4, ng/dl	1.14 ± 0.2
TSH ranges (µUI/ml)	
0.4-4, n (%)	65 (91)
>4, n (%)	4 (6)
<0.4, n (%)	2 (3)
Thyroid function	
Euthyroidism, n (%)	62 (87.3)
Hypothyroidism on treatment, n (%)	8 (4.1)
Hyperthyroidism on treatment, n (%)	1 (1.4)

TSH, thyroid stimulating hormone; FT3, free triiodothyronine; FT4, free thyroxine.

Biochemical and elastographic liver parameters are presented in **Table 4**. Regarding ELF score and the consequent risk of fibrosis, the mean value was 9.21 ± 1. Only 2 patients (3%) were at high risk, and then referred to the hepatologist, 19 patients (27%) were at moderate risk, and the remainder (70%) were at low risk. About elastographic parameters, the LSM mean value was 5.20 ± 2.3 kPa, while CAP mean value was 252.93 ± 51.3 dB/m. Only nine patients had LSM ≥7 kPa and five had LSM ≥8 kPa.

**Table 4:** Liver parameters [n (%), mean ± SD or median (IQR)].

Liver parameters	All subjects
Biochemical liver markers	
AST, U/L	17 (13-24)
ALT, U/L	30 (26-40)
γ-GT, U/L	31 (17-54)
Total bilirubin, mg/dL	0.64 (0.5-0.72)
Direct bilirubin, mg/dL	0.17 (0.14-0.21)
Albumin, g/dL	3.7 ± 0.3
ELF and components	
ELF score	9.21 ± 1
TIMP 1, ng/ml	150.32 ± 46.7
PIIINP, ng/ml	6.49 (5.02-8.64)
HA, ng/ml	70.21 (35.94-112.51)
Liver steatosis and fibrosis scores	
HSI	43.37 (40.5-48.46)
NAFLD-LFS	0.14 ± 1.4
FIB-4	1.28 (0.89-1.7)
NFS	0 ± 0.9
Elastographic parameters	
LSM, kPa	5.20 ± 2.3
LSM IQR/M, %	14.13 ± 5.4
CAP, dB/m	252.93 ± 51.3
LSM ≥7 kPa, n (%)	9 (13)
LSM ≥8 kPa n (%)	5 (7)
ELF score categories	
<9.8, n (%)	49 (70)
9.8-11.3, n (%)	19 (27)
>11.3, n (%)	2 (3)

AST, aspartate aminotransferase; ALT, alanine aminotransferase; γ-GT, γ-glutamyl transferase; ELF, enhanced liver fibrosis; TIMP1, tissue inhibitor of metalloproteinases 1; PIIINP, N-terminal propeptide of type III procollagen; HA, hyaluronic acid; HSI, hepatic steatosis index; NAFLD-LFS, non-alcoholic fatty liver disease – liver fat score; FIB-4, fibrosis-4 index; NFS, non-alcoholic fatty liver disease fibrosis score; LSM, liver stiffness measurement; IQR, interquartile range; M, median; CAP, controlled attenuation parameter.

### 3.2 Main results

The results of the univariate analysis are presented in **Table 5**.

**Table 5:** Correlations between ELF score and main demographic and clinical variables.

Variables	ELF Score	
	r	p
Demographic and anthropometric characteristics		
Age	0.43	**0.0002**
Sex (male = 1)	-0.01	0.9223
BMI, kg/m^2^	-0.32	**0.0069**
Waist circumference, cm	-0.18	0.1587
Hip circumference, cm	-0.31	**0.0123**
Organ(s) transplanted and outcomes		
Kidney (yes = 1)	-0.03	0.791
Liver (yes = 1)	0.00	0.9983
Graft rejection history (yes = 1)	0.08	0.5155
Time since transplantation, months	0.21	0.0923
Immunosuppressive therapy		
Tacrolimus (yes = 1)	-0.33	**0.0056**
Mycophenolate (yes = 1)	-0.38	**0.0013**
Steroid (yes = 1)	0.03	0.7931
Cyclosporin (yes = 1)	0.17	0.1676
Everolimus (yes = 1)	0.36	**0.0029**
Therapy		
Metformin (yes = 1)	-0.20	0.0984
Sulfonylureas (yes = 1)	0.05	0.7101
SGLT2-i (yes = 1)	-0.06	0.6349
GLP1RA (yes = 1)	-0.30	**0.0141**
Fast-acting insulin (yes = 1)	-0.02	0.8697
Long-acting insulin (yes = 1)	-0.05	0.7115
Thyroid parameters		
TSH, µUI/ml	0.00	0.9859
FT3, pg/ml	-0.45	**0.0003**
FT4, ng/dl	-0.05	0.6891
Hepatic elastographic parameters		
LSM, kPa	0.09	0.4437
LSM ≥7 kPa (yes = 1)	0.15	0.2148
LSM ≥8 kPa (yes = 1)	0.08	0.5036
CAP, dB/m	-0.29	**0.0171**
Biochemical parameters		
Glycaemia, mg/dl	-0.17	0.1736
Glycated haemoglobin, mmol/mol	-0.16	0.1892
C-peptide, ng/mL	0.13	0.3143
Creatinine, mg/dL	0.39	**0.0009**
AST, U/L	0.5	**< 0.0001**
ALT, U/L	0.33	**0.0059**
Albumin, g/dL	-0.38	**0.0024**
LDL cholesterol, mg/dL	-0.2	0.1096
Triglycerides, mg/dL	-0.11	0.3853
Haemoglobin, g/dL	-0.39	**0.0009**

BMI, body mass index; SGLT2-i, sodium-glucose cotransporter-2 inhibitor; DPP4-i, dipeptidyl peptidase-4 inhibitor; GLP1RA, glucagon-like peptide-1 receptor agonist; TSH, thyroid stimulating hormone; FT3, free triiodothyronine; FT4, free thyroxine; LSM, liver stiffness measurement; CAP, controlled attenuation parameter; AST, aspartate aminotransferase; ALT, alanine aminotransferase; LDL, low-density lipoprotein. Bold values indicate statistically significant p-values.

**Figure 1:**
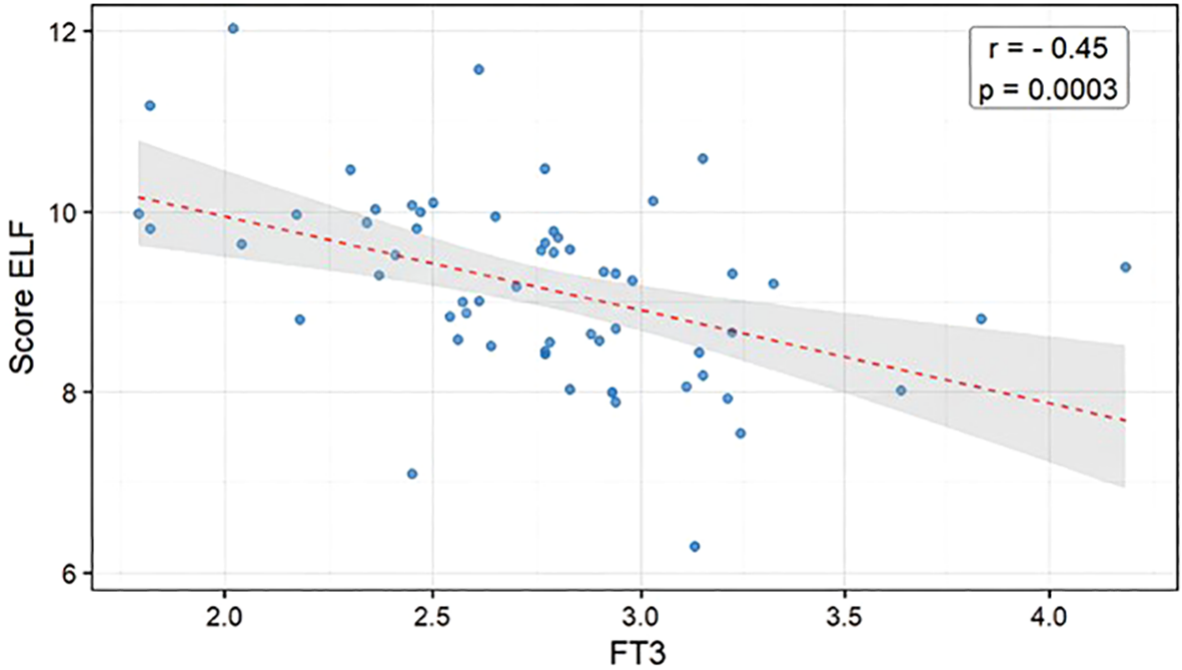
Relationship between enhanced liver fibrosis score (ELF) and free triiodothyronine (FT3) Inverse and statistically significant relationship (r = –0.45, p = 0.0003) between FT3 and ELF score. Blue dots represent individual patients; the red dashed line indicates the linear regression between FT3 and ELF score; the grey area represents the 95% confidence interval of the regression. r = Pearson’s correlation coefficient between ELF score and FT3; p = p-value for the significance of the linear regression.

ELF score was strongly and inversely associated with free triiodothyronine (FT3) (r = –0.45, p = 0.0003) (**Figure 1**), while no association was found between ELF score and TSH or FT4 (r=0.00, p=0.9859; r=-0.5, p=0.6891). All three components of the ELF score (TIMP1, PIIINP, and HA) were also significantly and inversely associated with FT3, as shown in **Supplementary Table S1**, **Supplementary Figure S1**.

Given the hepatology-focused aim of the study, we also performed dedicated subgroup analyses comparing liver versus non-liver transplant recipients to more specifically assess the impact of liver transplantation. When liver-transplant recipients were excluded, the inverse association between ELF and FT3 remained consistent with the main results (r = –0.45, p = 0.0029). When the analysis was restricted to liver-transplant recipients only, a similar trend was observed, although statistical significance was not reached due to the small sample size (r = –0.40, p = 0.0898). Importantly, even after excluding levothyroxine-treated participants, the inverse association between FT3 and ELF remained significant (r = –0.45, p = 0.0003).

Several other metabolic parameters were found to be significantly associated with ELF score, including age (r = 0.43; p = 0.0002); BMI (r = -0.32; p = 0.0069), hip circumference (r = -0.31; p = 0.0123), AST (r = 0.50; p = <0.0001), ALT (r = 0.33; p = 0.0059), and serum creatinine (r = 0.39; p = 0.0009). Haemoglobin and albumin were significantly inversely associated with ELF score (r = -0.39; p = 0.0009 and r = -0.38; p = 0.0024, respectively). Among transplantation-related variables, a significant association was found between ELF score and tacrolimus administration (r = –0.33, p = 0.0056), mycophenolate (r = –0.38, p = 0.0013), and everolimus (r = 0.36, p = 0.0029). The only non-immunosuppressive therapy that showed a correlation with ELF was GLP1-RA therapy (r = -0.3; p = 0.0141).

Neither post-transplant duration nor graft-rejection history showed any correlation with ELF values. No association was observed between LSM at VCTE and ELF score, whereas CAP was significantly and inversely associated with ELF score (r = –0.29, p = 0.0171). When LSM was analyzed as a categorical variable (≥7 or ≥8 kPa), no statistically significant association with ELF was observed, although a consistent trend toward higher ELF values was seen among patients with increased LSM.

In the multivariate model, after adjustment for age, sex, BMI, transaminases, glycated haemoglobin, creatinine, haemoglobin, albumin, immunosuppressive therapy, transplanted organ type and GPL-1RA treatment (R2 = 0.67 P = 0.0002), only age (p = 0.03) and FT3 (p = 0.04) remained significantly and independently associated with ELF score (**Table 6**, **Supplementary Figure S2**).

**Table 6:** Correlations between ELF score and main demographic and clinical variables after multivariate regression.

Analysis	ELF score		
	R²	β (SE)	p
Overall model	0.67		0.0002
FT3		-0.611 (0.288)	**0.0404**
Age		0.029 (0.012)	**0.0304**
BMI		-0.023 (0.029)	0.4266
Sex (male = 1)		-0.529 (0.438)	0.1376
Tacrolimus (yes = 1)		-0.401 (0.468)	0.3979
Mycophenolate (yes = 1)		-0.218 (0.291)	0.4577
Everolimus (yes = 1)		-0.331 (0.549)	0.5501
GLP1RA therapy (yes = 1)		-0.255 (0.305)	0.4076
AST		0.021 (0.017)	0.2266
ALT		-0.006 (0.019)	0.7580
Creatinine		0.143 (0.251)	0.5725
Glycated hemoglobin		-0.002 (0.019)	0.9162
Hemoglobin		-0.091 (0.086)	0.2987
Albumin		-0.064 (0.525)	0.9036
Kidney transplant (yes = 1)		-0.722 (0.737)	0.3334
Liver transplant (yes = 1)		-0.676 (0.679)	0.3260
Heart transplant (yes = 1)		-0.750 (0.768)	0.3350
Pancreas transplant (yes = 1)		0.141 (0.682)	0.8372
Lung transplant (yes = 1)		0.475 (1.175)	0.6880

FT3, free triiodothyronine. BMI, body mass index. GLP1RA, glucagon-like peptide-1 receptor agonist. AST, aspartate aminotransferase. ALT, alanine aminotransferase. Bold values indicate statistically significant p-values.

To evaluate the impact of glycaemic and pharmacological adjustments, sensitivity analyses excluding HbA1c and/or GLP-1RA were performed (**Supplementary Table S2**). In these models, the association between FT3 and ELF increased in magnitude while remaining directionally consistent.

## 4 Discussion

To our knowledge, this is the first study in SOT recipients with diabetes or prediabetes showing that lower FT3 levels, within the biochemical euthyroid range, are independently associated with a higher ELF score. ELF score is a biomarker that directly reflects extracellular matrix (ECM) turnover and predicts liver-related outcomes. Previous investigations of thyroid function and liver fibrosis have shown that low-normal thyroid function, as well as subclinical or overt hypothyroidism, is associated with an increased risk of liver fibrosis across multiple populations. However, these studies have primarily relied on surrogate non-invasive indices such as FIB-4, NFS, or VCTE (21, 40–42).

The relationship between thyroid function and liver fibrosis is complex and conflicting evidence has been reported in the literature. Some studies focusing on euthyroid adults have shown that higher FT3 levels are associated with an increased risk of steatosis and fibrosis, potentially reflecting compensatory metabolic adaptations or altered TH sensitivity (43, 44). However, there is extensive literature relating low-normal thyroid function, as well as subclinical or overt hypothyroidism, to an increased risk of liver fibrosis across multiple populations. Bano et al. conducted a meta-analysis, demonstrating that in the general population both overt and subclinical hypothyroidism are associated with a significantly increased risk of liver fibrosis (40). Specifically, this risk was 2–3 times higher compared to euthyroid individuals, with a dose-dependent relationship between elevated TSH levels and fibrosis. Fan et al. analysed 19,946 adults (mean age 47 years) who underwent abdominal ultrasound and thyroid function tests, dividing them into groups with strictly normal thyroid function, low-normal thyroid function, and subclinical hypothyroidism (41). Both low-normal thyroid function and subclinical hypothyroidism were found to be associated with an increased risk of MASLD and, among patients with MASLD, with a significantly higher risk of advanced hepatic fibrosis (defined as FIB-4 >2.67 and/or NFS > 0.676). In Martínez-Escudé et al. study, low-normal thyroid function (defined as higher TSH with normal FT3 and FT4 levels) was associated with increased liver stiffness and a higher prevalence of hepatic fibrosis compared with individuals with strictly normal thyroid function (42). After adjustment for metabolic confounders, the risk of fibrosis remained elevated, although statistical significance was attenuated. Specifically concerning FT3, previous evidence has shown that low FT3 is independently linked to advanced fibrosis, suggesting a protective role of sufficient TH activity. Manka et al., in a cohort of 144 adult outpatients at high risk of fibrosis and with a high prevalence of comorbidities such as obesity, diabetes and metabolic syndrome, found an association between low FT3 levels and liver fibrosis, as assessed by LSM and the NAFLD Fibrosis Score, even after adjustment for the main metabolic parameters and liver function indices (45).

TH, especially T3, play a key role in regulating hepatic lipid metabolism, mitochondrial activity, and key inflammatory/fibrogenic pathways; reduced FT3 levels may contribute to fibrosis progression. These findings confirm that subtle reductions in biologically active FT3 may reflect or contribute to pro-fibrogenic signalling by promoting hepatic inflammation and impairing the resolution of liver injury (19, 29, 45, 46).

It remains challenging to determine whether low FT3 levels could be a cause of liver fibrosis or a consequence of it. Low FT3 levels as a consequence of liver fibrosis is plausible, indeed advanced fibrosis, similar to other chronic conditions, can lead to non-thyroidal illness syndrome (NTIS), characterized by reduced peripheral conversion of T4 to T3, resulting in low FT3 levels despite normal TSH and FT4. Supporting this, evidence shows that low FT3 correlates with markers of disease severity and frailty in chronic liver disease as well as in other systemic diseases (45, 47–49). Conversely, the role of low FT3 as a causal factor in liver fibrosis development is supported by observational and mechanistic evidence.

Several lines of evidence support a biologically plausible, immune-mediated link between low FT3 and hepatic fibrosis. Injury-related activation of Hedgehog signalling induces type-3 deiodinase (DIO3) expression in the liver, thereby reducing intrahepatic T3 availability despite normal serum TSH and FT4 levels and altering hepatic metabolism and mitochondrial function. This mechanism establishes a feed-forward loop in which local thyroid-hormone deprivation further amplifies fibrogenic signalling (30, 50).

In addition, thyroid hormones shape macrophage and Kupffer-cell phenotype as well as their inflammatory output. Adequate T3 restrains nuclear factor-κB (NF-κB)-driven inflammatory responses and promotes resolution-associated functions, whereas reduced thyroid-hormone signalling skews macrophages toward pro-inflammatory states that release cytokines as key drivers of hepatic stellate-cell activation and extracellular matrix deposition (51, 52).

In summary, the association between low FT3 and liver fibrosis is likely bidirectional: low FT3 may both contribute to and result from progressive hepatic fibrosis, mediated by effects on hepatic metabolism, mitochondrial function, inflammation, and fibrogenesis, as well as reflecting the severity of underlying liver dysfunction. This may establish a vicious cycle, whereby reduced FT3 levels promote hepatic fibrosis, which in turn further suppresses FT3 concentrations, ultimately contributing to the progression and worsening of fibrosis.

Our findings do not support a significant modulation of thyroid–liver signaling by the immunosuppressive milieu but align with the concept of “intrahepatic hypothyroidism” in fibrosis, well documented in the general population and preserved in SOT recipients, despite profound metabolic and pharmacological perturbations. Furthermore, our data are consistent with the hypothesis that hepatic thyroid signalling deficiency may be relevant even when standard thyroid indices appear normal (20, 40, 45). Unlike studies conducted in the general population, in our cohort TSH did not correlate with fibrosis markers. This may reflect both biological and methodological aspects: TSH is primarily regulated by central pituitary feedback and can remain within the normal range despite peripheral alterations in hepatic TH metabolism, whereas FT3 more directly reflects T4-to-T3 conversion and intrahepatic thyroid hormone availability (19, 28, 31). In addition, all participants were solid organ transplant recipients with diabetes or prediabetes, representing a population exposed to dysmetabolic drivers and already at uniformly high metabolic risk.

Notably, in our cohort, thyroid parameters did not show any significant association with VCTE-based elastographic measures, including LSM and CAP. A plausible explanation is that LSM does not behave as a linear, continuous marker of fibrogenic activity: within the normal or low-intermediate range the metric carries limited information about ongoing fibrogenic activity (53, 54).

In the same analysis exploring correlates of ELF score, LSM was not significantly associated, whereas CAP was inversely associated with ELF score. This may reflect the different nature of the tests: ELF integrates dynamic ECM turnover (HA, PIIINP, TIMP-1) and captures fibrogenic activity, while LSM is influenced by additional factors such as vascular congestion, inflammation, and intrahepatic pressure (8, 55). Since CAP quantifies steatosis and ELF reflects fibrosis, the inverse correlation likely mirrors the natural history of MASLD, where loss of steatosis often accompanies fibrosis progression (56–58).

The present study has some limitations that must be acknowledged. First, the single-centre design and sample size limit precision; however, this was partially counterbalanced by the extensive and detailed analysis performed. Second, the cohort was heterogeneous, including patients with different transplanted organs and, in some cases, multiple transplants. We acknowledge that liver-transplant recipients represent a biologically distinct subgroup, as pre-existing hepatic fibrosis and graft-specific factors may influence post-transplant ELF levels and thyroid–liver interactions. Nevertheless, the study was designed to investigate thyroid–liver crosstalk across the full spectrum of solid-organ recipients exposed to comparable metabolic and immunosuppressive environments rather than to address graft-specific outcomes. To minimize potential bias, both the type of transplanted organ and the immunosuppressive regimen were incorporated into the multivariable models, and all patients were clinically and immunologically stable at the time of inclusion. Third, a subset of patients had prediabetes rather than overt diabetes. However, in the SOT setting, prediabetes frequently coexists with insulin resistance and treatment-related metabolic stressors and therefore confers a cardiometabolic risk profile that substantially overlaps with established diabetes. Although glycaemic control (HbA1c) and GLP-1RA therapy could lie on the causal pathway linking thyroid function to liver outcomes, they were included in the primary models as key metabolic confounders in this high-risk, pharmacologically complex population. To ensure that this choice did not mask thyroid-related signals, supplementary sensitivity analyses excluding HbA1c and/or GLP-1RA were performed and confirmed consistent effect direction with larger estimates in reduced models. Fourth, patients receiving levothyroxine therapy were not excluded, as this represents a frequent and clinically relevant phenotype in transplant medicine. Because LT4 provides T4 and circulating FT3 depends on peripheral T4-to-T3 conversion, modulated in the liver by deiodinase activity, serum FT3 remains a valid marker of functional thyroid hormone availability when therapy and thyroid tests are stable, as in our cohort. Fifth, although all patients were clinically stable, organ-specific factors and immunosuppression intensity may still confound residual risk. Nevertheless, the primary focus of this study was on liver alterations, as all enrolled patients had evidence of hepatic involvement (either steatosis or elevated FIB-4 index), which provides a common ground for the interpretation of the results. Finally, the absence of correlation between ELF and LSM, likely due to limited fibrosis prevalence and sample size, may restrict generalisability. This limitation is further amplified by the highly selected study population, which required hepatic involvement and diabetes/prediabetes, as well as by the very small number of less frequent transplant types, including lung and combined organ recipients.

## 5 Conclusion

Overall, our findings suggest a mechanistic link between lower FT3 levels and fibrogenic activity, as captured by ELF, in metabolically vulnerable transplant recipients. SOT recipients represent a clinically relevant model to test whether thyroid–liver associations observed in the general population are preserved in a setting of chronic immunosuppression and metabolic risk shaped by graft-adapted immune regulation. These findings support the relevance of FT3 in liver risk stratification after transplantation. Further studies are required to clarify causal direction, better characterize fibrogenic pathways in this population, and determine whether modulation of thyroid hormone signaling may have clinical utility in selected high-risk SOT recipients. In this context, resmetirom, a selective TH receptor-β agonist with proven efficacy in MASLD/MASH, could be evaluated in clinical trials targeting transplant recipients with low-normal FT3 levels and increased risk of liver fibrosis.

## Data Availability

The raw data supporting the conclusions of this article will be made available by the authors, without undue reservation.
