# Supplementary material for: Thyroid function and liver fibrosis: FT3 levels are inversely and independently correlated with enhanced liver fibrosis score in solid organ transplant patients with dysglycemia
Source: Front Immunol. 2026 Jan 8;16:1726617. doi: 10.3389/fimmu.2025.1726617 (PMC12823537; doi:10.3389/fimmu.2025.1726617)
Supplement: Supplementary file 1 [file DataSheet1.docx]

**SUPPLEMENTARY INFORMATION**

**Thyroid function and liver fibrosis: FT3 levels are inversely and independently correlated with enhanced liver fibrosis score in solid organ transplant patients with dysglycemia.**

*Claudia Leanza, Maria Ausilia Giusti, Vitale Miceli, Giovanni Zito, Rosaria Tinnirello, Gioacchin Iannolo, Antonio Galante, Fabrizio Emanuele, Marco Amato, Giovanna Lo Iacono, Vincenzina Lo Re, Salvatore Gruttadauria, Aldo Eugenio Calogero, Massimo Pinzani and Alessandro Mattina*

| **Table S1.** Correlations between FT3 and hepatic parameters. | | | |
| --- | --- | --- | --- |
|  |  | **FT3** | |
|  |  | **r** | **p** |
| **ELF and components** |  |  |  |
|  | ELF score | 0.45 | **0.0003** |
|  | TIMP 1, ng/ml | -0.45 | **0.0003** |
|  | PIIINP, ng/ml | -0.4 | **0.0016** |
|  | HA, ng/ml | -0.36 | **0.0049** |
| **Liver steatosis and fibrosis scores** |  |  |  |
|  | HSI | 0.13 | 0.3156 |
|  | NAFLD-LFS | 0.28 | 0.0549 |
|  | FIB-4 | -0.14 | 0.2903 |
|  | NFS | 0.03 | 0.8529 |
| **Hepatic elastographic parameters** |  |  |  |
|  | LSM, kPa | -0.07 | 0.5709 |
|  | CAP, dB/m | 0.18 | 0.1676 |
| **Biochemical parameters** |  |  |  |
|  | AST, U/L | -0.23 | 0.0786 |
|  | ALT, U/L | -0.14 | 0.2884 |
|  | γ-GT, U/L | 0.02 | 0.8769 |
|  | Total bilirubin, mg/dL | 0.14 | 0.2724 |
|  | Direct bilirubin, mg/dL | 0.04 | 0.7883 |
|  |  |  |  |
| ELF: enhanced liver fibrosis; TIMP1: tissue inhibitor of metalloproteinases 1; PIIINP: N-terminal propeptide of type III procollagen; HA: hyaluronic acid; HSI: hepatic steatosis index; NAFLD-LFS: non-alcoholic fatty liver disease – liver fat score; FIB-4: fibrosis-4 index; NFS: non-alcoholic fatty liver disease fibrosis score; LSM: liver stiffness measurement; CAP: controlled attenuation parameter; AST: aspartate aminotransferase; ALT: alanine aminotransferase; γ-GT: γ-glutamyl transferase. | | | |

**Figure S1. Relationship between ELF Score components and FT3**

**
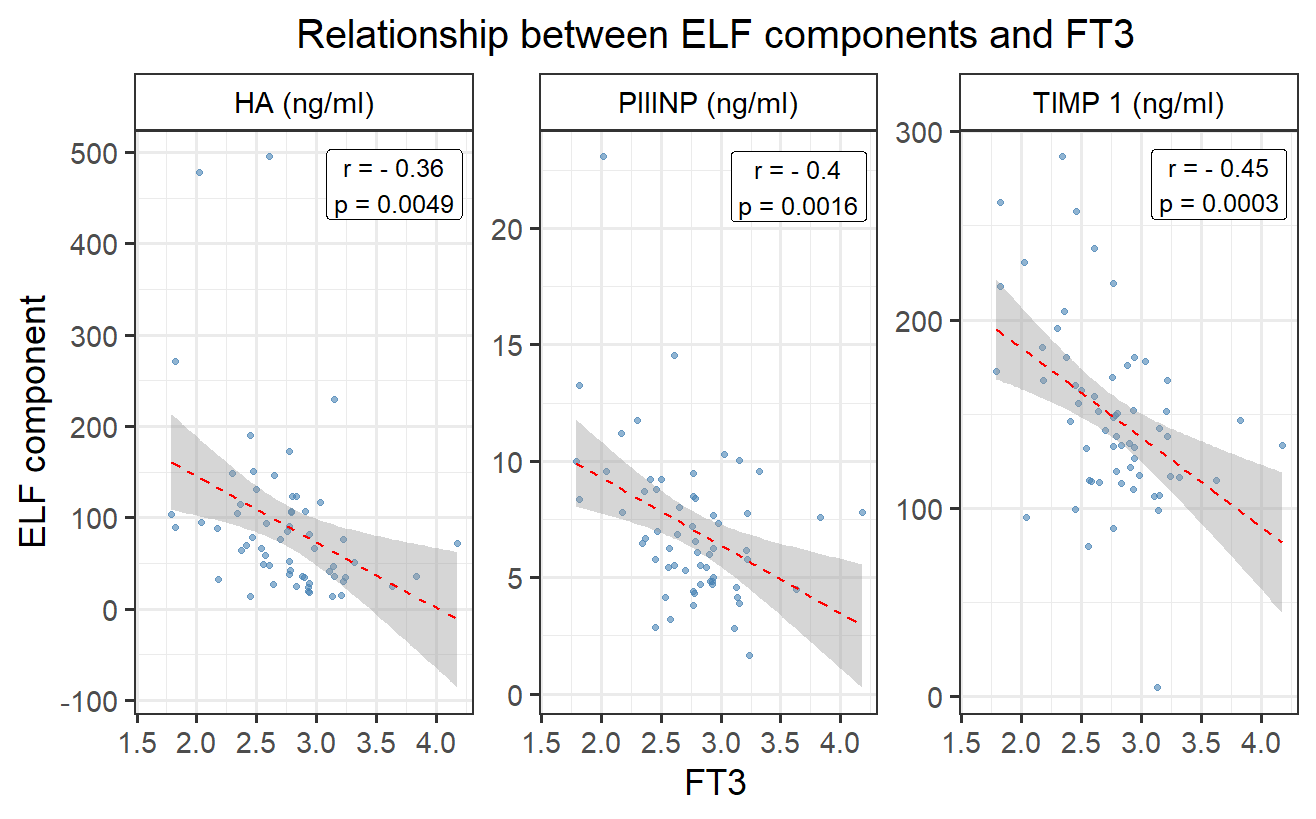
**

Inverse and statistically significant relationships between FT3 and the individual components of the ELF score (HA, PIIINP, and TIMP-1), with correlation coefficients of r = –0.36, –0.40, and –0.45, respectively (p = 0.0049, 0.0016, and 0.0003, respectively). For each plot: blue dots represent individual patients; the red dashed line indicates the linear regression between FT3 and the corresponding ELF component; the grey area represents the 95% confidence interval of the regression. HA: Hyaluronic Acid; PIIINP: Procollagen type III N-terminal Propeptide; TIMP-1: Tissue Inhibitor of Metalloproteinases-1; r = Pearson’s correlation coefficient between ELF component and FT3; p = p-value for the significance of the linear regression.

**Figure S2. Multivariate linear regression model**

**
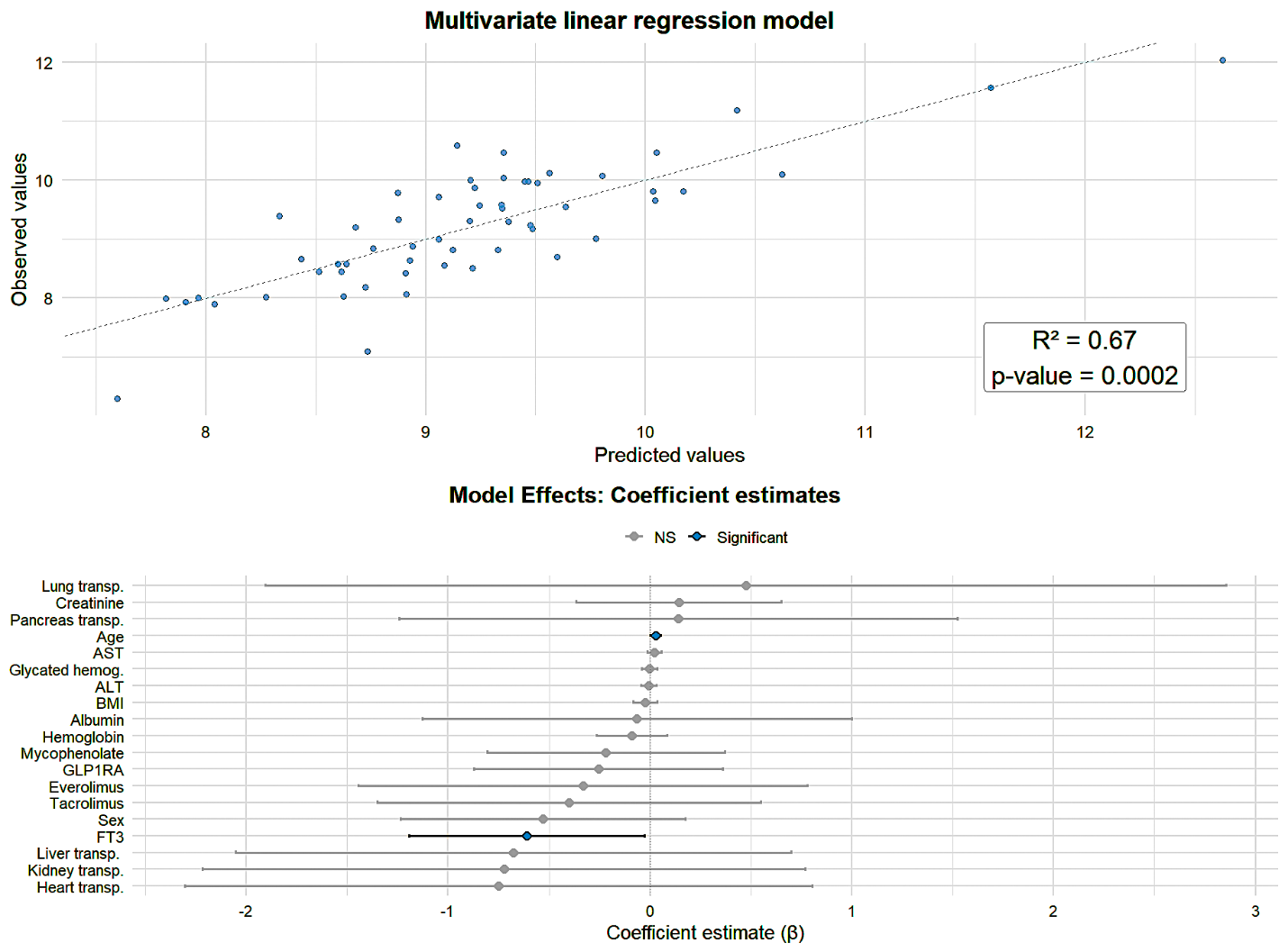
**

**Upper panel**: Comparison between observed ELF score values in the study population and those predicted by the multivariate linear regression model. Each blue dot represents the observed–predicted value pair for an individual patient; the diagonal dashed line indicates the line of perfect fit, i.e., where predicted and observed values coincide. The overall distribution of points indicates a good fit of the multivariate model to the data. R²: coefficient of determination of the model; p: p-value for the overall model significance. **Lower panel**: Estimated regression coefficients (β) of the variables included in the multivariate model with their corresponding 95% confidence intervals. Blue points indicate predictor variables showing a statistically significant effect on the ELF score.
